# Supplementary material for: Sweetened beverages and risk of frailty among older women in the Nurses’ Health Study: A cohort study
Source: PLoS Med. 2020 Dec 8;17(12):e1003453. doi: 10.1371/journal.pmed.1003453 (PMC7723265; doi:10.1371/journal.pmed.1003453)
Supplement: S1 Table — (DOCX) [file pmed.1003453.s001.docx]

| **S1** **Table**. Relative risks (95% confidence interval) of frailty according to categories of the most recent information of sweetened beverages consumption before the onset of frailty, among 67,739 women. | | | | | | | | |
| --- | --- | --- | --- | --- | --- | --- | --- | --- |
|  | Never or  almost  never | 1/mo to 3/mo | 1/wk | 2 to 6/wk | 1-2/d | ≥2/d | P for trend | Per 1 serving/d increase |
| **Sugar-sweetened beverages** | | |  |  |  | |  |  |
| Participants, n | 27,476 | 13,809 | 9655 | 12,616 | 3191 | 992 |  |  |
| Person-yr | 501,302 | 141,832 | 119,141 | 139,203 | 43,043 | 20,409 |  |  |
| Frailty cases, n | 5813 | 1417 | 1161 | 1608 | 495 | 312 |  |  |
| Age-adjusted | 1.00 | 1.01 (0.96, 1.07) | 1.08 (1.01, 1.15) | 1.24 (1.17, 1.32) | 1.33 (1.21, 1.46) | 1.82 (1.62, 2.04) | <0.001 | 1.24 (1.20, 1.28) |
| Multivariable model^a^ | 1.00 | 1.01 (0.95, 1.07) | 1.04 (0.97, 1.11) | 1.17 (1.10, 1.24) | 1.18 (1.08, 1.30) | 1.50 (1.34, 1.69) | <0.001 | 1.15 (1.12, 1.19) |
| Multivariable model^b^ | 1.00 | 0.99 (0.92, 1.05) | 1.00 (0.94, 1.07) | 1.11 (1.05, 1.18) | 1.11 (1.01, 1.22) | 1.39 (1.24, 1.57) | <0.001 | 1.12 (1.09, 1.16) |
| Multivariable model^c^ | 1.00 | 0.99 (0.93, 1.05) | 1.01 (0.95, 1.08) | 1.12 (1.05, 1.18) | 1.12 (1.02, 1.23) | 1.40 (1.25, 1.57) | <0.001 | 1.13 (1.09, 1.16) |
| **Artificially-sweetened beverages** | | |  |  |  |  |  |  |
| Participants, n | 21,153 | 7464 | 7129 | 18,334 | 8707 | 4952 |  |  |
| Person-yr | 462,042 | 96,250 | 96,219 | 182,701 | 77,507 | 50,208 |  |  |
| Frailty cases, n | 5396 | 1035 | 931 | 1868 | 879 | 697 |  |  |
| Age-adjusted | 1.00 | 1.09 (1.02, 1.17) | 1.09 (1.02, 1.17) | 1.26 (1.19, 1.32) | 1.60 (1.49, 1.72) | 2.24 (2.06, 2.43) | <0.001 | 1.27 (1.24, 1.29) |
| Multivariable model^a^ | 1.00 | 1.00 (0.93, 1.07) | 0.95 (0.89, 1.02) | 1.01 (0.96, 1.07) | 1.15 (1.07, 1.24) | 1.32 (1.21, 1.43) | <0.001 | 1.10 (1.07, 1.13) |
| Multivariable model^b^ | 1.00 | 0.99 (0.93, 1.06) | 0.95 (0.88, 1.02) | 1.00 (0.95, 1.05) | 1.12 (1.04, 1.21) | 1.27 (1.16, 1.38) | <0.001 | 1.09 (1.06, 1.11) |
| Multivariable model^c^ | 1.00 | 0.99 (0.93, 1.06) | 0.95 (0.88, 1.02) | 0.99 (0.94, 1.05) | 1.11 (1.03, 1.19) | 1.24 (1.14, 1.34) | <0.001 | 1.08 (1.05, 1.11) |
| **Total fruit juices** |  | |  |  |  | |  |  |
| Participants, n | 6895 | 6963 | 8068 | 23,918 | 18,889 | 3006 |  |  |
| Person-yr | 186,907 | 83,229 | 103,748 | 245,194 | 288,518 | 57,334 |  |  |
| Frailty cases, n | 2313 | 1007 | 1167 | 2733 | 2996 | 590 |  |  |
| Age-adjusted | 1.00 | 1.08 (1.00, 1.16) | 1.07 (0.99, 1.14) | 1.00 (0.94, 1.05) | 0.92 (0.87, 0.97) | 0.95 (0.87, 1.04) | <0.001 | 0.95 (0.92, 0.97) |
| Multivariable model^a^ | 1.00 | 1.02 (0.95, 1.10) | 1.02 (0.95, 1.10) | 1.00 (0.95, 1.06) | 0.95 (0.89, 1.00) | 0.99 (0.90, 1.09) | 0.10 | 0.98 (0.95, 1.00) |
| Multivariable model^b^ | 1.00 | 1.01 (0.94, 1.09) | 1.02 (0.95, 1.09) | 1.00 (0.94, 1.06) | 0.94 (0.89, 1.00) | 0.99 (0.90, 1.09) | 0.10 | 0.98 (0.95, 1.00) |
| Multivariable model^c^ | 1.00 | 1.01 (0.94, 1.09) | 1.01 (0.94, 1.09) | 1.00 (0.94, 1.06) | 0.94 (0.89, 0.99) | 0.99 (0.90, 1.09) | 0.10 | 0.98 (0.95, 1.00) |
|  | Never or  almost  never | 1/mo to 3/mo | 1/wk | 2 to 6/wk | ≥1/d |  |  | Per 1 serving/d increase |
| **Orange juice** |  |  |  |  |  |  |  |  |
| Participants, n | 13,568 | 12,041 | 8113 | 20,943 | 13,074 |  |  |  |
| Person-yr | 296,759 | 117,917 | 85,834 | 210,784 | 253,635 |  |  |  |
| Frailty cases, n | 3809 | 1312 | 909 | 2266 | 2510 |  |  |  |
| Age-adjusted | 1.00 | 1.05 (0.99, 1.12) | 0.97 (0.90, 1.04) | 0.91 (0.86, 0.96) | 0.83 (0.79, 0.88) |  | <0.001 | 0.87 (0.84, 0.90) |
| Multivariable model^a^ | 1.00 | 1.02 (0.96, 1.09) | 0.94 (0.87, 1.01) | 0.94 (0.89, 0.99) | 0.87 (0.83, 0.92) |  | <0.001 | 0.91 (0.87, 0.94) |
| Multivariable model^b^ | 1.00 | 1.01 (0.95, 1.08) | 0.93 (0.87, 1.00) | 0.93 (0.88, 0.98) | 0.86 (0.82, 0.91) |  | <0.001 | 0.90 (0.87, 0.94) |
| Multivariable model^c^ | 1.00 | 1.01 (0.95, 1.07) | 0.93 (0.86, 1.00) | 0.93 (0.88, 0.98) | 0.86 (0.82, 0.91) |  | <0.001 | 0.90 (0.87, 0.94) |
| **Other juices^*^** |  |  |  |  |  |  |  |  |
| Participants, n | 20,198 | 14,716 | 12,162 | 16,385 | 4278 |  |  |  |
| Person-yr | 449,341 | 148,077 | 138,367 | 157,871 | 71,273 |  |  |  |
| Frailty cases, n | 5178 | 1598 | 1324 | 1831 | 875 |  |  |  |
| Age-adjusted | 1.00 | 1.03 (0.97, 1.08) | 1.00 (0.94, 1.06) | 1.10 (1.04, 1.17) | 1.15 (1.06, 1.23) |  | <0.001 | 1.09 (1.04, 1.14) |
| Multivariable model^a^ | 1.00 | 1.00 (0.95, 1.06) | 0.99 (0.93, 1.06) | 1.10 (1.04, 1.17) | 1.16 (1.08, 1.25) |  | <0.001 | 1.10 (1.05, 1.14) |
| Multivariable model^b^ | 1.00 | 1.01 (0.95, 1.07) | 1.01 (0.94, 1.07) | 1.13 (1.07, 1.19) | 1.18 (1.10, 1.27) |  | <0.001 | 1.11 (1.07, 1.16) |
| Multivariable model^c^ | 1.00 | 1.01 (0.95, 1.06) | 1.00 (0.94, 1.07) | 1.13 (1.06, 1.19) | 1.18 (1.09, 1.27) |  | <0.001 | 1.11 (1.06, 1.16) |
| ^a^ Adjusted for: age (years), calendar time (4-y intervals), body mass index (<25.0, 25.0-29.9, ≥30.0 kg/m^2^), smoking status (never, past, and current 1-14, 15-24, and ≥25  cigarettes/day), alcohol intake (0, 1.0-4.9, 5.0-14.9, or ≥15.0 g/d), energy intake (quintiles of kcal/d), physical activity (quintiles) and medication use (aspirin, postmenopausal  hormone therapy, diuretics, β-blockers, calcium channel blockers, ACE inhibitors, other blood pressure medication, statins and other cholesterol lowering drugs, insulin, oral  hypoglycemic medication). ^b^ Adjusted for variables in model a and additionally adjusted for the Alternate Healthy Eating Index (quartiles). ^c^ Adjusted for variables in model b and additionally adjusted for cancer, heart disease and diabetes (yes/no). All beverages were mutually adjusted for each other. ^*^ This group includes apple juice or cider, grapefruit juice, prune juice, and non-specified fruit juices. | | | | | | | | |
