# Supplementary material for: Sweetened beverages and risk of frailty among older women in the Nurses’ Health Study: A cohort study
Source: PLoS Med. 2020 Dec 8;17(12):e1003453. doi: 10.1371/journal.pmed.1003453 (PMC7723265; doi:10.1371/journal.pmed.1003453)
Supplement: S2 Table — (DOCX) [file pmed.1003453.s002.docx]

| **S2** **Table**. Relative risks (95% confidence interval) of frailty according to categories of sweetened beverages consumption among 57,760 women with 0 frailty criteria at baseline. | | | | | | | | |
| --- | --- | --- | --- | --- | --- | --- | --- | --- |
|  | Never or  almost  never | 1/mo to 3/mo | 1/wk | 2 to 6/wk | 1-2/d | ≥2/d | P for trend | Per 1 serving/d increase |
| **Sugar-sweetened beverages** | | |  |  |  | |  |  |
| Participants, n | 23,173 | 12,031 | 8280 | 10,813 | 2679 | 784 |  |  |
| Person-yr | 304,628 | 203,326 | 132,924 | 173,012 | 33,955 | 7812 |  |  |
| Frailty cases, n | 2660 | 1839 | 1333 | 1669 | 363 | 71 |  |  |
| Age-adjusted | 1.00 | 1.00 (0.94, 1.06) | 1.10 (1.03, 1.18) | 1.16 (1.09, 1.23) | 1.58 (1.41, 1.76) | 1.79 (1.41, 2.26) | <0.001 | 1.31 (1.24, 1.38) |
| Multivariable model^a^ | 1.00 | 1.02 (0.96, 1.09) | 1.11 (1.03, 1.18) | 1.11 (1.04, 1.19) | 1.40 (1.26; 1.58) | 1.32 (1.03, 1.67) | <0.001 | 1.18 (1.11, 1.24) |
| Multivariable model^b^ | 1.00 | 1.00 (0.94, 1.06) | 1.07 (0.99, 1.14) | 1.05 (0.98, 1.12) | 1.29 (1.15; 1.45) | 1.20 (0.94, 1.52) | <0.001 | 1.12 (1.06, 1.19) |
| Multivariable model^c^ | 1.00 | 1.01 (0.95, 1.07) | 1.07 (1.00, 1.15) | 1.06 (0.99, 1.13) | 1.31 (1.16; 1.46) | 1.19 (0.94, 1.52) | <0.001 | 1.12 (1.06, 1.19) |
| **Artificially-sweetened beverages** | | |  |  |  |  |  |  |
| Participants, n | 17,995 | 6476 | 6182 | 15,841 | 7265 | 4001 |  |  |
| Person-yr | 269,332 | 121,639 | 98,449 | 240,634 | 86,537 | 39,067 |  |  |
| Frailty cases, n | 2278 | 1153 | 927 | 2281 | 861 | 435 |  |  |
| Age-adjusted | 1.00 | 1.07 (0.99, 1.15) | 1.14 (1.06, 1.23) | 1.31 (1.23, 1.39) | 1.73  (1.59, 1.87) | 2.38 (2.14, 2.64) | <0.001 | 1.29 (1.26, 1.32) |
| Multivariable model^a^ | 1.00 | 1.00 (0.93, 1.07) | 1.01 (0.93, 1.09) | 1.07 (1.01, 1.14) | 1.22 (1.13, 1.33) | 1.38 (1.24, 1.54) | <0.001 | 1.12 (1.09, 1.15) |
| Multivariable model^b^ | 1.00 | 1.01 (0.94, 1.08) | 1.01 (0.93, 1.09) | 1.06 (1.00, 1.13) | 1.20 (1.10, 1.30) | 1.32 (1.19, 1.48) | <0.001 | 1.11 (1.07, 1.14) |
| Multivariable model^c^ | 1.00 | 1.01 (0.93, 1.08) | 1.00 (0.93, 1.08) | 1.06 (1.00, 1.12) | 1.19 (1.09, 1.29) | 1.30 (1.16, 1.45) | <0.001 | 1.10 (1.07, 1.13) |
| **Total fruit juices** |  | |  |  |  | |  |  |
| Participants, n | 5656 | 5901 | 6883 | 20,760 | 16,077 | 2483 |  |  |
| Person-yr | 62,154 | 83,046 | 94,557 | 351,369 | 234,552 | 29,979 |  |  |
| Frailty cases, n | 553 | 800 | 916 | 3484 | 1983 | 199 |  |  |
| Age-adjusted | 1.00 | 0.94 (0.84, 1.05) | 0.90 (0.81, 1.00) | 0.83 (0.76, 0.91) | 0.78 (0.71, 0.86) | 0.77 (0.65, 0.90) | <0.001 | 0.89 (0.85, 0.93) |
| Multivariable model^a^ | 1.00 | 0.96 (0.86, 1.07) | 0.92 (0.83, 1.03) | 0.90 (0.82, 0.99) | 0.87 (0.78, 0.95) | 0.88 (0.75, 1.04) | 0.01 | 0.95 (0.91, 0.99) |
| Multivariable model^b^ | 1.00 | 0.95 (0.86, 1.06) | 0.92 (0.83, 1.02) | 0.90 (0.82, 0.99) | 0.87 (0.79, 0.96) | 0.90 (0.76, 1.06) | 0.02 | 0.96 (0.92, 1.00) |
| Multivariable model^c^ | 1.00 | 0.96 (0.86, 1.07) | 0.92 (0.83, 1.03) | 0.91 (0.83, 0.99) | 0.87 (0.79, 0.96) | 0.90 (0.76, 1.06) | 0.01 | 0.96 (0.92, 1.00) |
|  | Never or  almost  never | 1/mo to 3/mo | 1/wk | 2 to 6/wk | ≥1/d |  |  | Per 1 serving/d increase |
| **Orange juice** |  |  |  |  |  |  |  |  |
| Participants, n | 11,221 | 10,291 | 7008 | 18,216 | 11,024 |  |  |  |
| Person-yr | 139,402 | 136,843 | 108,675 | 327,024 | 143,713 |  |  |  |
| Frailty cases, n | 1366 | 1189 | 1084 | 3214 | 1082 |  |  |  |
| Age-adjusted | 1.00 | 0.89 (0.82, 0.96) | 0.87 (0.80, 0.94) | 0.81 (0.75, 0.86) | 0.77 (0.71, 0.83) |  | <0.001 | 0.85 (0.80, 0.89) |
| Multivariable model^a^ | 1.00 | 0.90 (0.83, 0.98) | 0.89 (0.82, 0.97) | 0.86 (0.80, 0.92) | 0.81 (0.75, 0.88) |  | <0.001 | 0.90 (0.86, 0.95) |
| Multivariable model^b^ | 1.00 | 0.90 (0.83, 0.97) | 0.89 (0.82, 0.96) | 0.86 (0.80, 0.91) | 0.81 (0.74, 0.88) |  | <0.001 | 0.90 (0.85, 0.95) |
| Multivariable model^c^ | 1.00 | 0.90 (0.83, 0.98) | 0.89 (0.82, 0.97) | 0.86 (0.80, 0.92) | 0.81 (0.74, 0.88) |  | <0.001 | 0.90 (0.85, 0.95) |
| **Other juices^*^** |  |  |  |  |  |  |  |  |
| Participants, n | 16,812 | 12,665 | 10,520 | 14,223 | 3540 |  |  |  |
| Person-yr | 217,611 | 210,743 | 163,635 | 226,544 | 37,124 |  |  |  |
| Frailty cases, n | 1942 | 2041 | 1556 | 2152 | 244 |  |  |  |
| Age-adjusted | 1.00 | 1.02 (0.96, 1.08) | 0.99 (0.92, 1.06) | 1.04 (0.97, 1.11) | 1.01 (0.88, 1.15) |  | 0.47 | 0.99 (0.92, 1.06) |
| Multivariable model^a^ | 1.00 | 1.05 (0.99, 1.12) | 1.03 (0.96, 1.10) | 1.11 (1.04, 1.18) | 1.05 (0.92, 1.21) |  | 0.05 | 1.04 (0.97, 1.12) |
| Multivariable model^b^ | 1.00 | 1.06 (1.00, 1.13) | 1.05 (0.97, 1.12) | 1.14 (1.07, 1.22) | 1.10 (0.96, 1.26) |  | 0.003 | 1.08 (1.01, 1.17) |
| Multivariable model^c^ | 1.00 | 1.06 (1.00, 1.13) | 1.04 (0.97, 1.12) | 1.14 (1.07, 1.22) | 1.09 (0.95, 1.25) |  | 0.004 | 1.08 (1.00, 1.16) |
| ^a^ Adjusted for: age (years), calendar time (4-y intervals), body mass index (<25.0, 25.0-29.9, ≥30.0 kg/m^2^), smoking status (never, past, and current 1-14, 15-24, and ≥25  cigarettes/day), alcohol intake (0, 1.0-4.9, 5.0-14.9, or ≥15.0 g/d), energy intake (quintiles of kcal/d), physical activity (quintiles) and medication use (aspirin, postmenopausal  hormone therapy, diuretics, β-blockers, calcium channel blockers, ACE inhibitors, other blood pressure medication, statins and other cholesterol lowering drugs, insulin, oral  hypoglycemic medication). ^b^ Adjusted for variables in model a and additionally adjusted for the Alternate Healthy Eating Index (quartiles). ^c^ Adjusted for variables in model b and additionally adjusted for cancer, heart disease and diabetes (yes/no). All beverages were mutually adjusted for each other. ^*^ This group includes apple juice or cider, grapefruit juice, prune juice, and non-specified fruit juices. | | | | | | | | |
