# Supplementary material for: Sweetened beverages and risk of frailty among older women in the Nurses’ Health Study: A cohort study
Source: PLoS Med. 2020 Dec 8;17(12):e1003453. doi: 10.1371/journal.pmed.1003453 (PMC7723265; doi:10.1371/journal.pmed.1003453)
Supplement: S3 Table — (DOCX) [file pmed.1003453.s003.docx]

| **S3** **Table**. Relative risks (95% confidence interval) of frailty according to categories of sweetened beverages consumption among 82,430 women aged ≥60y in the Nurses’ Health Study, including diet before baseline in 1980, 1984 and 1986. | | | | | | | | |
| --- | --- | --- | --- | --- | --- | --- | --- | --- |
|  | Never or  almost  never | 1/mo to 3/mo | 1/wk | 2 to 6/wk | 1-2/d | ≥2/d | P for trend | Per 1 serving/d increase |
| **Sugar-sweetened beverages** | | |  |  |  | |  |  |
| Participants, n | 23,666 | 21,043 | 13,929 | 19,442 | 3547 | 803 |  |  |
| Person-yr | 322,657 | 321,001 | 211,939 | 272,330 | 43,049 | 8165 |  |  |
| Frailty cases, n | 3237 | 3466 | 2358 | 3195 | 552 | 132 |  |  |
| Age-adjusted | 1.00 | 1.05 (1.00, 1.10) | 1.12 (1.06, 1.18) | 1.28 (1.22, 1.35) | 1.66 (1.51, 1.81) | 2.57 (2.16, 3.07) | <0.001 | 1.43 (1.37, 1.48) |
| Multivariable model^a^ | 1.00 | 1.03 (0.98, 1.08) | 1.06 (1.01, 1.12) | 1.17 (1.11, 1.23) | 1.35 (1.23, 1.48) | 1.78 (1.49, 2.13) | <0.001 | 1.23 (1.18, 1.29) |
| Multivariable model^b^ | 1.00 | 1.01 (0.96, 1.06) | 1.02 (0.97, 1.08) | 1.10 (1.04, 1.16) | 1.24 (1.13, 1.36) | 1.62 (1.35, 1.93) | <0.001 | 1.18 (1.13, 1.23) |
| Multivariable model^c^ | 1.00 | 1.01 (0.96, 1.06) | 1.03 (0.97, 1.08) | 1.10 (1.04, 1.16) | 1.25 (1.13, 1.37) | 1.59 (1.33, 1.90) | <0.001 | 1.18 (1.12, 1.23) |
| **Artificially-sweetened beverages** | | |  |  |  |  |  |  |
| Participants, n | 21,533 | 11,430 | 9489 | 25,648 | 10,218 | 4112 |  |  |
| Person-yr | 314,546 | 181,571 | 148,766 | 366,176 | 124,522 | 43,560 |  |  |
| Frailty cases, n | 2975 | 1840 | 1663 | 4212 | 1630 | 620 |  |  |
| Age-adjusted | 1.00 | 1.03 (0.97, 1.09) | 1.21 (1.14, 1.28) | 1.37 (1.31, 1.44) | 1.83 (1.72, 1.95) | 2.27 (2.08, 2.48) | <0.001 | 1.36 (1.33, 1.40) |
| Multivariable model^a^ | 1.00 | 0.94 (0.89, 1.00) | 1.06 (1.00, 1.12) | 1.08 (1.03, 1.13) | 1.24 (1.16, 1.32) | 1.34 (1.22, 1.47) | <0.001 | 1.13 (1.11, 1.16) |
| Multivariable model^b^ | 1.00 | 0.95 (0.89, 1.00) | 1.07 (1.00, 1.13) | 1.08 (1.03, 1.14) | 1.23 (1.15, 1.31) | 1.32 (1.20, 1.44) | <0.001 | 1.13 (1.10, 1.15) |
| Multivariable model^c^ | 1.00 | 0.94 (0.89, 1.00) | 1.06 (1.00, 1.13) | 1.07 (1.02, 1.13) | 1.22 (1.14, 1.30) | 1.29 (1.18, 1.41) | <0.001 | 1.12 (1.09, 1.15) |
| **Total fruit juices** |  | |  |  |  | |  |  |
| Participants, n | 3588 | 7730 | 9715 | 40,286 | 18,934 | 2177 |  |  |
| Person-yr | 43,149 | 100,068 | 137,874 | 591,562 | 279,255 | 27,232 |  |  |
| Frailty cases, n | 485 | 1137 | 1552 | 6703 | 2852 | 211 |  |  |
| Age-adjusted | 1.00 | 0.93 (0.83, 1.03) | 0.87 (0.78, 0.96) | 0.85 (0.77, 0.93) | 0.77 (0.70, 0.85) | 0.67 (0.57, 0.79) | <0.001 | 0.88 (0.85, 0.91) |
| Multivariable model^a^ | 1.00 | 0.90 (0.81, 1.00) | 0.86 (0.78, 0.95) | 0.87 (0.79, 0.95) | 0.83 (0.75, 0.91) | 0.76 (0.65, 0.90) | <0.001 | 0.94 (0.91, 0.98) |
| Multivariable model^b^ | 1.00 | 0.90 (0.81, 1.00) | 0.86 (0.78, 0.96) | 0.87 (0.80, 0.96) | 0.84 (0.76, 0.93) | 0.79 (0.67, 0.93) | 0.01 | 0.96 (0.92, 0.99) |
| Multivariable model^c^ | 1.00 | 0.90 (0.81, 1.00) | 0.86 (0.78, 0.96) | 0.87 (0.79, 0.96) | 0.84 (0.76, 0.93) | 0.78 (0.66, 0.92) | 0.004 | 0.96 (0.92, 0.99) |
|  | Never or  almost  never | 1/mo to 3/mo | 1/wk | 2 to 6/wk | ≥1/d |  |  | Per 1 serving/d increase |
| **Orange juice** |  |  |  |  |  |  |  |  |
| Participants, n | 11,231 | 14,092 | 11,891 | 33,767 | 11,449 |  |  |  |
| Person-yr | 143,398 | 185,429 | 175,993 | 522,120 | 152,201 |  |  |  |
| Frailty cases, n | 1615 | 2050 | 2030 | 5831 | 1414 |  |  |  |
| Age-adjusted | 1.00 | 0.95 (0.89, 1.01) | 0.91 (0.86, 0.98) | 0.86 (0.81, 0.91) | 0.80 (0.75, 0.86) |  | <0.001 | 0.86 (0.82, 0.90) |
| Multivariable model^a^ | 1.00 | 0.93 (0.87, 0.99) | 0.91 (0.85, 0.97) | 0.87 (0.82, 0.93) | 0.83 (0.77, 0.89) |  | <0.001 | 0.90 (0.86, 0.94) |
| Multivariable model^b^ | 1.00 | 0.92 (0.86, 0.98) | 0.91 (0.85, 0.97) | 0.87 (0.82, 0.92) | 0.82 (0.77, 0.89) |  | <0.001 | 0.90 (0.86, 0.94) |
| Multivariable model^c^ | 1.00 | 0.92 (0.86, 0.98) | 0.91 (0.85, 0.97) | 0.87 (0.82, 0.92) | 0.82 (0.76, 0.89) |  | <0.001 | 0.90 (0.86, 0.94) |
| **Other juices^*^** |  |  |  |  |  |  |  |  |
| Participants, n | 17,682 | 20,406 | 16,490 | 24,046 | 3806 |  |  |  |
| Person-yr | 231,221 | 307,254 | 249,682 | 346,953 | 44,030 |  |  |  |
| Frailty cases, n | 2445 | 3560 | 2792 | 3771 | 372 |  |  |  |
| Age-adjusted | 1.00 | 1.02 (0.97, 1.07) | 0.99 (0.94, 1.05) | 1.01 (0.96, 1.06) | 0.95 (0.85, 1.06) |  | 0.43 | 0.95 (0.90, 1.01) |
| Multivariable model^a^ | 1.00 | 1.04 (0.99, 1.10) | 1.04 (0.98, 1.10) | 1.09 (1.03, 1.15) | 1.04 (0.93, 1.16) |  | 0.07 | 1.03 (0.97, 1.09) |
| Multivariable model^b^ | 1.00 | 1.05 (1.00, 1.11) | 1.06 (1.00, 1.12) | 1.12 (1.06, 1.19) | 1.06 (0.97, 1.22) |  | 0.001 | 1.08 (1.01, 1.14) |
| Multivariable model^c^ | 1.00 | 1.05 (1.00, 1.11) | 1.06 (1.00, 1.12) | 1.12 (1.06, 1.19) | 1.08 (0.97, 1.21) |  | 0.002 | 1.07 (1.01, 1.13) |

^a^ Adjusted for: age (years), calendar time (4-y intervals), body mass index (<25.0, 25.0-29.9, ≥30.0 kg/m^2^), smoking status (never, past, and current 1-14, 15-24, and ≥25

cigarettes/day), alcohol intake (0, 1.0-4.9, 5.0-14.9, or ≥15.0 g/d), energy intake (quintiles of kcal/d), physical activity (quintiles) and medication use (aspirin, postmenopausal

hormone therapy, diuretics, β-blockers, calcium channel blockers, ACE inhibitors, other blood pressure medication, statins and other cholesterol lowering drugs, insulin, oral

hypoglycemic medication). ^b^ Adjusted for variables in model a and additionally adjusted for the Alternate Healthy Eating Index (quartiles). ^c^ Adjusted for variables in model b and additionally adjusted for cancer, heart disease and diabetes (yes/no). All beverages were mutually adjusted for each other. ^*^ This group includes apple juice or cider, grapefruit juice, prune juice, and non-specified fruit juices.
