# Supplementary material for: Sweetened beverages and risk of frailty among older women in the Nurses’ Health Study: A cohort study
Source: PLoS Med. 2020 Dec 8;17(12):e1003453. doi: 10.1371/journal.pmed.1003453 (PMC7723265; doi:10.1371/journal.pmed.1003453)
Supplement: S4 Table — (DOCX) [file pmed.1003453.s004.docx]

| **S4** **Table**. Relative risks (95% confidence interval) of frailty according to sweetened beverage intake (serving/d) among women aged ≥60y in the Nurses’ Health Study. Latency analysis | | | | | |
| --- | --- | --- | --- | --- | --- |
|  | **Sugar-sweetened beverages** | **Artificially-sweetened beverages** | **Total fruit juices** | **Orange juice** | **Other juices^*^** |
| **Lagged 6 years** |  |  |  |  |  |
| Age-adjusted | 1.39 (1.32; 1.46) | 1.35 (1.32 1.38) | 0.86 (0.83; 0.90) | 0.84 (0.80; 0.88) | 0.95 (0.89; 1.01) |
| Multivariable model^a^ | 1.21 (1.15, 1.27) | 1.14 (1.11; 1.17) | 0.95 (0.91, 0.99) | 0.90 (0.86, 0.95) | 1.07 (1.00, 1.15) |
| Multivariable model^b^ | 1.14 (1.08; 1.20) | 1.13 (1.10; 1.16) | 0.97 (0.92, 1.01) | 0.90 (0.85, 0.94) | 1.12 (1.05, 1.20) |
| Multivariable model^c^ | 1.14 (1.08; 1.20) | 1.12 (1.09; 1.15) | 0.97 (0.93, 1.01) | 0.89 (0.85, 0.94) | 1.12 (1.05, 1.20) |
| **Lagged 8 years** |  |  |  |  |  |
| Age-adjusted | 1.35 (1.29; 1.42) | 1.33 (1.29; 1.36) | 0.87 (0.84; 0.90) | 0.86 (0.82; 0.90) | 0.93 (0.87; 0.99) |
| Multivariable model^a^ | 1.20 (1.14; 1.26) | 1.13 (1.10; 1.16) | 0.96 (0.92, 1.00) | 0.92 (0.87, 0.96) | 1.05 (0.98, 1.12) |
| Multivariable model^b^ | 1.13 (1.07; 1.18) | 1.12 (1.09; 1.15) | 0.97 (0.94, 1.01) | 0.92 (0.87, 0.96) | 1.09 (1.03, 1.16) |
| Multivariable model^c^ | 1.13 (1.07; 1.18) | 1.11 (1.08; 1.14) | 0.97 (0.93, 1.01) | 0.91 (0.87, 0.96) | 1.09 (1.03, 1.16) |
| **Lagged 12 years** |  |  |  |  |  |
| Age-adjusted | 1.31 (1.25; 1.36) | 1.30 (1.27; 1.33) | 0.88 (0.84; 0.91) | 0.87 (0.83; 0.91) | 0.93 (0.88; 0.99) |
| Multivariable model^a^ | 1.17 (1.12; 1.23) | 1.12 (1.09; 1.15) | 0.96 (0.92, 1.00) | 0.93 (0.89, 0.97) | 1.04 (0.98, 1.10) |
| Multivariable model^b^ | 1.11 (1.06; 1.16) | 1.11 (1.08; 1.14) | 0.98 (0.94, 1.02) | 0.93 (0.88, 0.97) | 1.08 (1.02, 1.15) |
| Multivariable model^c^ | 1.11 (1.06; 1.16) | 1.11 (1.08; 1.13) | 0.98 (0.94, 1.01) | 0.92 (0.88, 0.97) | 1.08 (1.02, 1.14) |
| ^a^ Adjusted for: age (years), calendar time (4-y intervals), body mass index (<25.0, 25.0-29.9, ≥30.0 kg/m^2^), smoking status (never, past, and current 1-14, 15-24, and ≥25  cigarettes/day), alcohol intake (0, 1.0-4.9, 5.0-14.9, or ≥15.0 g/d), energy intake (quintiles of kcal/d), physical activity (quintiles) and medication use (aspirin, postmenopausal  hormone therapy, diuretics, β-blockers, calcium channel blockers, ACE inhibitors, other blood pressure medication, statins and other cholesterol lowering drugs, insulin, oral  hypoglycemic medication). ^b^ Adjusted for variables in model a and additionally adjusted for the Alternate Healthy Eating Index (quartiles). ^c^ Adjusted for variables in model b and additionally adjusted for cancer, heart disease and diabetes (yes/no). All beverages were mutually adjusted for each other. ^*^ This group includes apple juice or cider, grapefruit juice, prune juice, and non-specified fruit juices. | | | | | |
