# Supplementary material for: Sweetened beverages and risk of frailty among older women in the Nurses’ Health Study: A cohort study
Source: PLoS Med. 2020 Dec 8;17(12):e1003453. doi: 10.1371/journal.pmed.1003453 (PMC7723265; doi:10.1371/journal.pmed.1003453)
Supplement: S5 Table — (DOCX) [file pmed.1003453.s005.docx]

| **S5** **Table**. Relative risks (95% confidence interval) of frailty according to categories of sweetened beverages consumption among 60,402 women aged ≥60y without heart disease, diabetes or cancer in the Nurses’ Health Study. | | | | | | | | |
| --- | --- | --- | --- | --- | --- | --- | --- | --- |
|  | Never or  almost  never | 1/mo to 3/mo | 1/wk | 2 to 6/wk | 1-2/d | ≥2/d | P for trend | Per 1 serving/d increase |
| **Sugar-sweetened beverages** | | |  |  |  | |  |  |
| Participants, n | 23,730 | 12,660 | 8718 | 11,510 | 2906 | 878 |  |  |
| Person-yr | 287,157 | 197,340 | 128,750 | 168,859 | 34,153 | 7977 |  |  |
| Frailty cases, n | 2288 | 1752 | 1249 | 1643 | 355 | 67 |  |  |
| Age-adjusted | 1.00 | 1.06 (0.99, 1.13) | 1.17 (1.09, 1.25) | 1.28 (1.19, 1.36) | 1.68 (1.50; 1.88) | 1.85 (1.45, 2.36) | <0.001 | 1.35 (1.28, 1.43) |
| Multivariable model^a^ | 1.00 | 1.02 (0.96, 1.09) | 1.10 (1.03, 1.19) | 1.16 (1.08, 1.24) | 1.41 (1.25; 1.58) | 1.37 (1.07, 1.75) | <0.001 | 1.19 (1.13, 1.26) |
| Multivariable model^b^ | 1.00 | 0.99 (0.93, 1.06) | 1.06 (0.98, 1.13) | 1.07 (1.00, 1.15) | 1.26 (1.12; 1.42) | 1.20 (0.94, 1.54) | <0.001 | 1.12 (1.06, 1.19) |
| **Artificially-sweetened beverages** | | |  |  |  |  |  |  |
| Participants, n | 19,188 | 6778 | 6385 | 16,407 | 7591 | 4053 |  |  |
| Person-yr | 263,475 | 118,030 | 93,401 | 230,137 | 82,850 | 36,345 |  |  |
| Frailty cases, n | 2166 | 1104 | 847 | 2150 | 739 | 348 |  |  |
| Age-adjusted | 1.00 | 1.08 (1.00, 1.16) | 1.14 (1.05, 1.23) | 1.32 (1.25, 1.41) | 1.59 (1.46, 1.73) | 2.11 (1.87, 2.36) | <0.001 | 1.31 (1.27, 1.35) |
| Multivariable model^a^ | 1.00 | 1.01 (0.94, 1.09) | 1.01 (0.93, 1.09) | 1.12 (1.05, 1.19) | 1.19 (1.09, 1.30) | 1.43 (1.27, 1.61) | <0.001 | 1.15 (1.11, 1.18) |
| Multivariable model^b^ | 1.00 | 1.01 (0.94, 1.09) | 1.01 (0.93, 1.09) | 1.10 (1.04, 1.17) | 1.15 (1.06, 1.26) | 1.36 (1.21, 1.53) | <0.001 | 1.12 (1.09, 1.16) |
| **Total fruit juices** |  | |  |  |  | |  |  |
| Participants, n | 5982 | 6347 | 7300 | 21,696 | 16,453 | 2624 |  |  |
| Person-yr | 60,235 | 83,108 | 92,315 | 339,172 | 220,337 | 29,070 |  |  |
| Frailty cases, n | 490 | 771 | 869 | 3315 | 1733 | 176 |  |  |
| Age-adjusted | 1.00 | 0.96 (0.85, 1.07) | 0.93 (0.84, 1.04) | 0.86 (0.78, 0.95) | 0.76 (0.68, 0.84) | 0.71 (0.60, 0.85) | <0.001 | 0.86 (0.82, 0.90) |
| Multivariable model^a^ | 1.00 | 0.95 (0.85, 1.07) | 0.95 (0.85, 1.06) | 0.92 (0.83, 1.01) | 0.86 (0.77, 0.95) | 0.83 (0.70, 0.99) | <0.001 | 0.94 (0.89, 0.98) |
| Multivariable model^b^ | 1.00 | 0.94 (0.84, 1.06) | 0.94 (0.84, 1.06) | 0.92 (0.84, 1.02) | 0.86 (0.78, 0.96) | 0.85 (0.71, 1.01) | 0.003 | 0.94 (0.90, 0.99) |
|  | Never or  almost  never | 1/mo to 3/mo | 1/wk | 2 to 6/wk | ≥1/d |  |  | Per 1 serving/d increase |
| **Orange juice** |  |  |  |  |  |  |  |  |
| Participants, n | 11,829 | 11,062 | 7386 | 18,874 | 11,251 |  |  |  |
| Person-yr | 135,945 | 135,331 | 106,069 | 312,630 | 134,261 |  |  |  |
| Frailty cases, n | 1240 | 1183 | 1042 | 2983 | 906 |  |  |  |
| Age-adjusted | 1.00 | 0.94 (0.87, 1.02) | 0.91 (0.83, 0.99) | 0.83 (0.77, 0.88) | 0.73 (0.67, 0.80) |  | <0.001 | 0.81 (0.77, 0.86) |
| Multivariable model^a^ | 1.00 | 0.96 (0.88, 1.04) | 0.93 (0.86, 1.01) | 0.87 (0.81, 0.93) | 0.80 (0.73, 0.87) |  | <0.001 | 0.87 (0.82, 0.92) |
| Multivariable model^b^ | 1.00 | 0.95 (0.88, 1.03) | 0.93 (0.85, 1.01) | 0.87 (0.81, 0.93) | 0.79 (0.72, 0.86) |  | <0.001 | 0.86 (0.82, 0.91) |
| **Other juices^*^** |  |  |  |  |  |  |  |  |
| Participants, n | 17,672 | 13,426 | 10,872 | 14,694 | 3738 |  |  |  |
| Person-yr | 209,526 | 206,375 | 156,134 | 216,223 | 35,979 |  |  |  |
| Frailty cases, n | 1777 | 1944 | 1438 | 1968 | 227 |  |  |  |
| Age-adjusted | 1.00 | 1.00 (0.94, 1.07) | 0.97 (0.90, 1.04) | 1.00 (0.94, 1.07) | 0.97 (0.84, 1.12) |  | 0.82 | 0.97 (0.90, 1.05) |
| Multivariable model^a^ | 1.00 | 1.02 (0.95, 1.09) | 1.01 (0.94, 1.09) | 1.08 (1.01, 1.15) | 1.11 (0.96, 1.27) |  | 0.02 | 1.08 (1.00, 1.17) |
| Multivariable model^b^ | 1.00 | 1.03 (0.96, 1.10) | 1.03 (0.96, 1.11) | 1.13 (1.05, 1.21) | 1.16 (1.01, 1.34) |  | <0.001 | 1.13 (1.04, 1.21) |
| ^a^ Adjusted for: age (years), calendar time (4-y intervals), body mass index (<25.0, 25.0-29.9, ≥30.0 kg/m^2^), smoking status (never, past, and current 1-14, 15-24, and ≥25 cigarettes/day), alcohol intake (0, 1.0-4.9, 5.0-14.9, or ≥15.0 g/d), energy intake (quintiles of kcal/d), physical activity (quintiles) and medication use (aspirin, postmenopausal hormone therapy, diuretics, β-blockers, calcium channel blockers, ACE inhibitors, other blood pressure medication, statins and other cholesterol lowering drugs, insulin, oral hypoglycemic medication). ^b^ Additionally adjusted for the Alternate Healthy Eating Index (quartiles). All beverages were mutually adjusted for each other. ^*^ This group includes apple juice or cider, grapefruit juice, prune juice, and non-specified fruit juices. | | | | | | | | |
