# Supplementary material for: Sweetened beverages and risk of frailty among older women in the Nurses’ Health Study: A cohort study
Source: PLoS Med. 2020 Dec 8;17(12):e1003453. doi: 10.1371/journal.pmed.1003453 (PMC7723265; doi:10.1371/journal.pmed.1003453)
Supplement: S6 Table — (DOCX) [file pmed.1003453.s006.docx]

| **S6 Table**. Relative risks (95% confidence interval) of frailty with the weight loss criteria defined as a 10% weight reduction in 2 years according to categories of sweetened beverages consumption among 72,180 women aged ≥60y in the Nurses’ Health Study.^a^ | | | | | | | | |
| --- | --- | --- | --- | --- | --- | --- | --- | --- |
|  | Never or  almost  never | 1/mo to 3/mo | 1/wk | 2 to 6/wk | 1-2/d | ≥2/d | P for trend | Per 1 serving/d increase |
| **Sugar-sweetened beverages** | | |  |  |  | |  |  |
| Participants, n | 29,081 | 14,758 | 10,264 | 13,534 | 3459 | 1084 |  |  |
| Person-yr | 376,415 | 247,145 | 162,522 | 213,428 | 43,406 | 10,652 |  |  |
| Frailty cases, n | 3360 | 2204 | 1590 | 2137 | 453 | 111 |  |  |
| Age-adjusted | 1.00 | 0.97 (0.91, 1.02) | 1.07 (1.01, 1.14) | 1.19 (1.12, 1.25) | 1.49 (1.35, 1.65) | 1.93 (1.60, 2.34) | <0.001 | 1.32 (1.26, 1.38) |
| Multivariable model^b^ | 1.00 | 0.99 (0.94, 1.05) | 1.08 (1.01, 1.15) | 1.14 (1.07, 1.20) | 1.30 (1.18, 1.44) | 1.43 (1.18, 1.74) | <0.001 | 1.18 (1.12, 1.24) |
| Multivariable model^c^ | 1.00 | 0.97 (0.92, 1.03) | 1.04 (0.98, 1.10) | 1.07 (1.01 1.13) | 1.19 (1.08, 1.32) | 1.29 (1.06, 1.57) | <0.001 | 1.12 (1.07, 1.18) |
| Multivariable model^d^ | 1.00 | 0.98 (0.93, 1.04) | 1.05 (0.98, 1.11) | 1.08 (1.02, 1.15) | 1.21 (1.09, 1.34) | 1.29 (1.06, 1.57) | <0.001 | 1.13 (1.07, 1.18) |
| **Artificially-sweetened beverages** | | |  |  |  |  |  |  |
| Participants, n | 22,392 | 7902 | 7568 | 19,600 | 9360 | 5358 |  |  |
| Person-yr | 328,667 | 146,917 | 118,763 | 295,851 | 111,135 | 52,236 |  |  |
| Frailty cases, n | 2768 | 1376 | 1109 | 2853 | 1109 | 640 |  |  |
| Age-adjusted | 1.00 | 1.06 (0.99, 1.13) | 1.14 (1.06, 1.22) | 1.31 (1.25, 1.39) | 1.68 (1.56, 1.80) | 2.47 (2.26, 2.70) | <0.001 | 1.30 (1.27, 1.33) |
| Multivariable model^b^ | 1.00 | 1.00 (0.93, 1.06) | 1.01 (0.94, 1.08) | 1.08 (1.02, 1.14) | 1.16 (1.08, 1.25) | 1.40 (1.28, 1.54) | <0.001 | 1.12 (1.09, 1.14) |
| Multivariable model^c^ | 1.00 | 1.00 (0.94, 1.07) | 1.01 (0.94, 1.08) | 1.07 (1.02, 1.13) | 1.13 (1.05, 1.22) | 1.35 (1.23, 1.48) | <0.001 | 1.10 (1.08, 1.13) |
| Multivariable model^d^ | 1.00 | 1.00 (0.94, 1.07) | 1.00 (0.94, 1.08) | 1.07 (1.01, 1.13) | 1.12 (1.04, 1.21) | 1.32 (1.20, 1.44) | <0.001 | 1.09 (1.07, 1.12) |
| **Total fruit juices** |  | |  |  |  | |  |  |
| Participants, n | 7322 | 7551 | 8736 | 25,681 | 19,739 | 3151 |  |  |
| Person-yr | 79,336 | 105,099 | 118,143 | 429,870 | 283,689 | 37,431 |  |  |
| Frailty cases, n | 720 | 1033 | 1149 | 4280 | 2420 | 253 |  |  |
| Age-adjusted | 1.00 | 0.95 (0.86, 1.04) | 0.91 (0.82, 0.99) | 0.84 (0.77, 0.91) | 0.78 (0.72, 0.85) | 0.75 (0.65, 0.86) | <0.001 | 0.89 (0.85, 0.92) |
| Multivariable model^b^ | 1.00 | 0.96 (0.87, 1.06) | 0.93 (0.85, 1.02) | 0.92 (0.85, 1.00) | 0.88 (0.80, 0.96) | 0.87 (0.75, 1.00) | 0.002 | 0.95 (0.92, 0.99) |
| Multivariable model^c^ | 1.00 | 0.95 (0.86, 1.05) | 0.93 (0.84, 1.02) | 0.92 (0.85, 1.00) | 0.88 (0.81, 0.96) | 0.88 (0.76, 1.02) | 0.01 | 0.96 (0.92, 1.00) |
| Multivariable model^d^ | 1.00 | 0.96 (0.87, 1.06) | 0.93 (0.85, 1.02) | 0.93 (0.85, 1.01) | 0.88 (0.81, 0.96) | 0.88 (0.76, 1.01) | 0.004 | 0.96 (0.92, 0.99) |
|  | Never or  almost  never | 1/mo to 3/mo | 1/wk | 2 to 6/wk | ≥1/d |  |  | Per 1 serving/d increase |
| **Orange juice** |  |  |  |  |  |  |  |  |
| Participants, n | 14,437 | 13,081 | 8726 | 22,353 | 13,583 |  |  |  |
| Person-yr | 176,671 | 171,468 | 134,266 | 396,629 | 174,533 |  |  |  |
| Frailty cases, n | 1749 | 1576 | 1330 | 3867 | 1333 |  |  |  |
| Age-adjusted | 1.00 | 0.93 (0.87, 1.00) | 0.86 (0.80, 0.93) | 0.81 (0.76, 0.85) | 0.77 (0.71, 0.83) |  | <0.001 | 0.82 (0.78, 0.86) |
| Multivariable model^b^ | 1.00 | 0.95 (0.88, 1.01) | 0.91 (0.84, 0.97) | 0.88 (0.83, 0.93) | 0.82 (0.76, 0.88) |  | <0.001 | 0.89 (0.84, 0.93) |
| Multivariable model^c^ | 1.00 | 0.94 (0.88, 1.01) | 0.90 (0.84, 0.97) | 0.87 (0.82, 0.93) | 0.81 (0.76, 0.88) |  | <0.001 | 0.88 (0.84, 0.92) |
| Multivariable model^d^ | 1.00 | 0.95 (0.88, 1.01) | 0.91 (0.84, 0.98) | 0.88 (0.82, 0.93) | 0.81 (0.75, 0.87) |  | <0.001 | 0.88 (0.84, 0.92) |
| **Other juices^e^** |  |  |  |  |  |  |  |  |
| Participants, n | 21,423 | 15,805 | 13,002 | 17,454 | 4496 |  |  |  |
| Person-yr | 272,363 | 259,150 | 199,629 | 275,439 | 46,987 |  |  |  |
| Frailty cases, n | 2479 | 2523 | 1881 | 2608 | 364 |  |  |  |
| Age-adjusted | 1.00 | 1.01 (0.95, 1.06) | 0.97 (0.91, 1.03) | 1.01 (0.96, 1.07) | 1.11 (0.99, 1.24) |  | 0.10 | 1.03 (0.97, 1.10) |
| Multivariable model^b^ | 1.00 | 1.05 (0.99, 1.11) | 1.01 (0.95, 1.08) | 1.09 (1.02, 1.15) | 1.14 (1.02, 1.28) |  | 0.003 | 1.09 (1.02, 1.16) |
| Multivariable model^c^ | 1.00 | 1.05 (0.99, 1.11) | 1.03 (0.97, 1.10) | 1.12 (1.06, 1.19) | 1.19 (1.06, 1.33) |  | <0.001 | 1.12 (1.05, 1.19) |
| Multivariable model^d^ | 1.00 | 1.06 (1.00, 1.12) | 1.03 (0.97, 1.10) | 1.13 (1.06, 1.19) | 1.18 (1.05, 1.32) |  | <0.001 | 1.12 (1.05, 1.19) |
| ^a^ The number of participants is slightly higher compared to the main analysis because less persons were excluded due to frailty at baseline. ^b^Adjusted for: age (years), calendar time (4-y intervals), body mass index (<25.0, 25.0-29.9, ≥30.0 kg/m^2^), smoking status (never, past, and current 1-14, 15-24, and ≥25 cigarettes/day), alcohol intake (0, 1.0-4.9, 5.0-14.9, or ≥15.0 g/d), energy intake (quintiles of kcal/d), physical activity (quintiles) and medication use (aspirin, postmenopausal hormone therapy, diuretics, β-blockers, calcium channel blockers, ACE inhibitors, other blood pressure medication, statins and other cholesterol lowering drugs, insulin, oral hypoglycemic medication). ^c^ Adjusted for variables in model a and additionally adjusted for the Alternate Healthy Eating Index (quartiles). ^d^ Adjusted for variables in model b and additionally adjusted for cancer, heart disease and diabetes (yes/no). All beverages were mutually adjusted for each other. ^e^ This group includes apple juice or cider, grapefruit juice, prune juice, and non-specified fruit juices | | | | | | | | |
