# Supplementary material for: Sweetened beverages and risk of frailty among older women in the Nurses’ Health Study: A cohort study
Source: PLoS Med. 2020 Dec 8;17(12):e1003453. doi: 10.1371/journal.pmed.1003453 (PMC7723265; doi:10.1371/journal.pmed.1003453)
Supplement: Attachment — Submitted filename: Response to reviewers_PLOSMed.docx [file pmed.1003453.s008.docx]

**Requests from the editors:**

*Please revise your title according to PLOS Medicine's style - as well as setting out the study question this should also summarise the study design/framework used, after a colon (eg, ": prospective cohort).

The title has now been adjusted to: ´Sweetened beverages and risk of frailty among older women in the Nurses´ Health Study: a prospective cohort study´.

*At this stage, we ask that you include a short, non-technical Author Summary of your research to make findings accessible to a wide audience that includes both scientists and non-scientists. The Author Summary should immediately follow the Abstract in your revised manuscript. This text is subject to editorial change and should be distinct from the scientific abstract. Please see our author guidelines for more information: <https://journals.plos.org/plosmedicine/s/revising-your-manuscript#loc-author-summary>

The author summary has now been included in the manuscript. Please, see page 3-4.

*The abstract should have some minor changes to structure, please use the headings Background, Methods and Findings, Conclusions (nb, "Methods and Findings" is a single subsection).

The abstract has now been adjusted (Page 2).

*In the last sentence of the Abstract Methods and Findings section, please include a brief note about any key limitation(s) of the study's methodology.

The following text has been added to the end of the abstract Methods and Findings section.

Page 2*: ´A limitation of this study is that, due to self-reporting of diet and frailty, certain misclassification bias cannot be ruled-out; also, some residual confounding may persist´.*

*Did your study have a prospective protocol or analysis plan? Please state this (either way) early in the Methods section.

a) If a prospective analysis plan (from your funding proposal, IRB or other ethics committee submission, study protocol, or other planning document written before analyzing the data) was used in designing the study, please include the relevant prospectively written document with your revised manuscript as a Supporting Information file to be published alongside your study, and cite it in the Methods section. A legend for this file should be included at the end of your manuscript.

b) If no such document exists, please make sure that the Methods section transparently describes when analyses were planned, and when/why any data-driven changes to analyses took place.

c) In either case, changes in the analysis-- including those made in response to peer review comments-- should be identified as such in the Methods section of the paper, with rationale.

There was no prospective protocol or analysis plan for this analysis. We have included this in the Methods section, as well as the changes made in the analysis.

Page 7: ´*There was no formal prospectively written protocol for the current study. All analyses described below were decided a priori, except for the additional adjustment for physical activity and morbidity, which was suggested by the reviewers´.*

*It's encouraging the paper has been reported using the STROBE guideline - however we'd suggest also appending the completed STROBE checklist as a supporting information file alongside the submitted revised paper - checklist can be downloaded at https://www.equator-network.org/reporting-guidelines/strobe/. When completing the checklist, please use section and paragraph numbers, rather than page numbers.

We have now uploaded the STROBE checklist.

-----------------------------------------------------------

**Comments from the reviewers:**

**Reviewer #1:** In this manuscript, authors investigate the association of SSB, ASB and fruit juice intake and risk of frailty in women 60 years and older, using data from the Nurses' Health Study. They report a statistically significant increased risk of frailty with high consumption of SSB, ASB and fruit juice other than orange juice, and a lower risk of frailty with high consumption of orange juice. The manuscript is well written, and easy to read. There are number of strengths to this analysis: authors use a well-established sizable cohort with good quality data; prospective study design; long follow up; and repeated exposure measurement during follow up. They also conducted a comprehensive set of statistical analyses, including a number of sensitivity analyses. Nonetheless, physical activity variable notably misses from their multivariable models, despite being an important confounder (major limitation). They report from a stratified analysis by PA level (low/high), though this does not seem enough to rule out the confounding effect of PA. I suggest repeating all the models including METh/wk.

Thank you for your comments. The role of physical activity in the association between sweetened beverage consumption and frailty is complex due to its close relation with the outcome. We initially did not adjust for physical activity in the main analysis to prevent over adjustment. As suggested, we have now included baseline physical activity in all multivariable models; please see the new tables. The results did not materially change after this additional adjustment.

Table 3: it would be helpful to see p for interaction. Perhaps a footnote indicating a statistically significant interaction.

We have now included the p for interactions in Table 3 and added these results to the text.

Page 20: ´*We found a significant interaction for SSB and orange juice with age, however, the stratified results do not show large differences in estimates for women aged <70 compared to women aged ≥70*´.

-----------------------------------------------------------

**Reviewer #2**: Struijk et al. present a large prospective epidemiological study on sweetened beverages and risk for frailty. The study has indisputable strengths, including a very large sample size (n=almost 76,000 women), and a long follow-up (up to 22 years) between exposures (comprehensive exposure of most sweetened beverages) and ascertainment of frailty in older age. Very few studies on this topic have been conducted so far, therefore this report is important for the research field.

It is generally acknowledged that frailty has many risk factors with small to moderate effect sizes, and given the aging of the population worldwide, the observed decreased risk for frailty with higher orange juice intake could lead to a strong reduction in the number of cases.

In sum, the paper is well written overall although the way nutritional aspects are discussed should be improved. The authors would benefit from deciphering underlying mechanisms, instead of just mentioning obesity, diabetes, cardiovascular diseases or inflammation, and anti-oxidant properties of fruits to explain their results. A more complicated approach of the food matrix effect of fruit intakes compared with fruit juices intake would have been interesting. Moreover, a discussion about the possible reversibility of frailty would have reinforce the discussion section.

More specific remarks:

Abstract:

The "dose-response manner" mentioned in the conclusion is not ascertained by the retained results of the abstract. Please remove.

We have now removed these words from the Abstract and Discussion.

Introduction:

Are the artificially sweetened beverages (ASBs) largely consume among USA residents? The prevalence of consumption is only described for sugar-sweetened beverages SSBs and fruit juices.

Thank you, we have now included information about the consumption of artificially sweetened beverages in the Introduction.

Page 6: ´About 23.1% of women aged 60 and older in the U.S. population consumed ASBs on a given day during 2009‒2010 [Fakhouri et al, 2012]´.

A mention of the association between higher fruits intake or between carotenoids levels and the lower risk of frailty among European older people should be considered (Garcia-Esquinas et al 2016 and Pilleron et al 2019).

Thank you for these suggestions, we have now included both references.

Page 5: ´*Some specific components of the human diet, including fruit, proteins, and micronutrients are thought to decrease frailty risk when consumed in adequate amounts [5, Garcia-Esquinas, 2016; Pilleron, 2019] ´.*

Methods:

Regarding the exposure, could the cumulative consumption of SSBs, ASBs and fruit juices be a relevant analysis? Indeed, some people may be considered both as SSBs consumers, OR ASBs consumers, while they consumed both beverages. I wondered how this cumulative exposure could influence the results.

Our data in Table 1 showed that those in the highest category of SSB intake have a lower intake of ASBs compared to those in the lowest SSB quintile, and vice versa. We adjusted all models for the beverages which were not the exposure of interest, in order to obtain the estimates for the association of one specific beverage with frailty, independently of the other beverages.

We have now further explored the combined analysis of SSB and ASB intakes on the risk of frailty. Results showed that a high intake of both beverages simultaneously had a detrimental effect on frailty (please see table below). We are now including this result in the manuscript.

Page 17: Joint analyses showed that higher consumptions of both beverages simultaneously had also a direct association with frailty, in comparison with the lowest consumption of both beverages [RR for higher tertile vs. lowest tertile: 1.18 (1.08, 1.29)]

|  | Tertiles of SSB consumption | | |
| --- | --- | --- | --- |
|  | Low | Intermediate | High |
| Tertiles of ASB consumption |  |  |  |
| Low, n participants | 9079 | 10,618 | 15,486 |
|  | 1.00 (Reference) | 0.98 (0.90, 1.07) | 1.02 (0.94, 1.11) |
| Intermediate, n participants | 4604 | 4161 | 3978 |
|  | 0.99 (0.91, 1,08) | 0.98 (0.90, 1.07) | 1.05 (0.96, 1.14) |
| High, n participants | 12,196 | 6706 | 5107 |
|  | 1.05 (0.97, 1.14) | 1.06 (0.97, 1.16) | 1.18 (1.08, 1.29) |

Since SSB and ASB have opposite effects than orange juice, we have preferred not to combine these consumptions.

Regarding the identification of incident frail participants, one out of five criteria is weight loss. I'm surprised to observe that the threshold retained was a reduction of 5% of weight during the last 2 years. How participants lost to follow-up for a visit were classified? -the delay between 2 visits being higher than 2 years? Moreover, why the authors have not considered the GLIM criteria (Cederholm et al 2018) to approach undernutrition (the consensus of experts proposed a reduction of weight of 5% for less than 6 months or 10% for 6 months and over).

The weight loss criterion in a participant that did not fill out the weight information in one of the bi-annual questionnaires was defined as missing. At baseline 7.3% of the women had a missing on the weight loss component of frailty. However, if the participant had three other frail criteria, this person could still be defined as frail.

We agree with the reviewer that the weight loss definition in the GLIM criteria could be a good option, but we have simply aimed to adhere to the original FRAIL scale, which defines weight loss as a ≥5% reduction within the past year. Since we had bi-annual data only, we adjusted this definition to our available information. We have now acknowledged this adjustment to the original score in the Methods.

We have conducted a sensitivity analysis in which we defined weight loss as a 10% weight reduction in 2 years. This resulted in a reduction in the number of persons identified as frail (n=9855), however the associations remained the same. This indicates that the adjustment does not bias the results.

Page 9: ´*Finally, because weight of the participants was available only biannually, the weight loss criterion was defined as a ≥5% decrease in the weight reported in a two-year period before the assessment of frailty´*.

Regarding frailty as a whole, it's acknowledged that subjective criteria are used, but it's surprising not to considered physical activity (MET are available) and a more objective scale than the FRAIL one (which also need a clinical judgment form the practitioner which seems not available here). The authors should acknowledge that the scale is a proxy of a more subjective scale and a misclassification could occur. Finally, the concordance between the FRAIL scale and the Fried criteria has been described in a care setting, but not yet among community-dwellers. This is another limitation.

We used the FRAIL scale because it is based on self-reported data, which is useful for large cohort studies based on information provided by the participants, such as the Nurses’ Health Study. The FRAIL scale somewhat considers physical activity within the item on low aerobic capacity, estimated by the ability to walk several hundred yards. In the Nurses’ Health Study, we have approximated this by the ability to walk several blocks or several miles, based on the available information.

According to the reviewer’s comment, we have now acknowledged in the Limitations section that misclassification could occur due to the use of subjective measurements of frailty. Additionally, we have specified that the correlation between the FRAIL scale and the Fried criteria was found in the care-setting.

Page 27: ´*Finally, performance-based measures were not available in this large cohort of older women. Due to the use of self-reported information, some misclassification of frailty might have occurred. Our results should be confirmed in studies using other frailty definitions that include more objective measurements [2, 4] ´.*

Page 9-10: ´*Despite the absence of performance-based measures, the FRAIL scale has been shown to be correlated with the Fried scale (r= 0.617, p<0.001) [28], the most widely used scale for frailty assessment, which includes both self-reported and performance-based measures, among older adults in care settings*´.

More importantly, how were considered participants with frailty and disability (in instrumental activities of daily living IADL): it may occur an overlap between both frailty and IADL disability, while some authors argued that frailty is not disability (and the scale used to identify frail people should also not identify IADL disabled participants, see Zamudio-Rodríguez A et al, Age Ageing 2020). This important point should be discussed in depths.

We agree that frailty and disability are different concepts, that frailty usually precedes disability although they may coexist in the same patient [Zamudio-Rodríguez et al, 2020]. Since disability is not available in this cohort, we cannot discard that in some participants frailty and IADL disability coexist. Therefore, future research should evaluate the effect of sweetened beverages separately on disability. Notwithstanding this, some previous research has shown that several diet-related factors (e.g., Mediterranean diet, fruits and vegetables) that lower the risk of frailty [Struijk et al, 2020; Kojima et al, 2018] also lower the risk of disability [Struijk et al, 2018; Féart et al, 2011; Artaud, 2013].

We have now included information on the relation between frailty and disability in the Discussion.

Page 26: *´Frailty is an important outcome because it is the consequence of alterations in many physiological systems. Frailty and pre-frail status as defined by the FRAIL scale has shown to be a significant predictor of disability among older adults [Kojima, 2018]. Although reverting frailty development is challenging for many patients, at an early stage frailty might be reversible and is therefore a valuable tool to identify those at risk for further adverse health effects [Zamudio-Rodríguez et al, 2020].* Some previous research has shown that several diet-related factors (e.g., Mediterranean diet, fruits and vegetables) that lower the risk of frailty [Struijk et al, 2020; Kojima et al, 2018] also lower the risk of disability [Féart et al, 2011; Artaud, 2013]. Thus, we could speculate that that sweetened beverages might also have a detrimental effect on disability.”

Regarding the statistical approach, I identified 2 major limits: the first one is the potential reversibility of the frailty status. It's acknowledged that frailty might be transitory (even more when the duration of the follow-up is high), and then, the identification of frail people who are then censured at the first time they are identified frail could induce a bias. The second limit is the lack of consideration of the risk for death: however, when the duration of the study is so long, the proportion of older people who died during the follow-u is high, and in the present study, the exposure can also be associated with this specific risk of death. Therefore, the analysis is mainly appropriate among the survivors, and the competitive risk for death compared with frailty cannot be dismissed. To limit this bias, an illness-death analysis should be performed. It would reinforce the results.

We agree that frailty is a dynamic condition and is potentially reversible. We have assessed frailty recovery in this cohort and we observed that, once frailty occurred it is not very likely to reverse (please see table below). Also, given that the goal was to assess the association between beverages consumption and risk of frailty among women without frailty, we censored those participants who developed frailty for the first time at follow-up. We have now included this in the Limitations section of the Discussion.

| Recovery at 4 y | 14.3% |
| --- | --- |
| Recovery at 8 y | 5.7% |
| Recovery at 12 y | 1.0% |

Page 27: ‘*Frailty is a dynamic condition and therefore, potentially reversible. However, as well as other chronic conditions, once it occurs, it is unlikely to reverse*.’

As regards the second limitation, our analyses used cause-specific proportional hazards models, which censored participants at their time of death, and account for competing risks. We have now made it explicit in the text.

Page 11: ´*We used cause-specific proportional hazards models to calculate relative risks (RR)* [Wang et al, 2016]´.

Results.

Table 1 provides the description of low, moderate or high consumers of specific beverages, while the statistical part provided data on 6 levels of consumption: how were these 6 different levels rearranged on 3 different levels of intake?

Table 1 presents only 3 levels of all consumption levels (aiming to indicate lowest, mid and highest intake) to make the table easier to read. We agree that these headings could be confusing, so we have now deleted them and only the specific levels of consumption are shown (Never or almost never, 1/wk, ≥2/d).

Table 1: is alcohol expressed as g/d? please specify.

Thank you for pointing this out. This should be ´g/d´ and now has been corrected.

The text relative to the description of Table 2 (and others) would benefit from details (for instance, 3 different RR are provided, but it's not easy to understand which exposure is linked to which RR).

Thank you, we have now improved the text. Please, see the new Results section.

The test of interaction (p value) between exposure, BMI … should be provided to ensure the relevance of such stratified analysis.

We have now included the p for interaction in Table 3 and added a line summarizing the results.

Page 20: ´*We found a significant interaction for SSB and orange juice with age, however, the stratified results do not show large differences in estimates for women aged <70 compared to women aged ≥70*.´.

Discussion.

In addition to the limits already discussed above, the relevance of such results among women only should be addressed.

We have now included this in the Limitations section of the Discussion.

Page 27: ´*Third,* a*lthough studying the risk of frailty among only female nurses helped to increase internal validity, the observed associations might not apply to other populations´.*

The discussion about the differences observed between orange juice or other fruits juices intake appears limited and only in the field of cognition. This should be discussed further, because of the public health relevance of such results. I suggest revising with deeper discussion on nutritional aspects.

The possible underlying mechanisms of the association between juices and frailty have now been discussed in more depth. We have added the following text to the Discussion section:

Page 25: ´*Many antioxidant nutrients and bioactive substances (including vitamins, carotenoids, flavonoids, and polyphenols) are found in juices, and especially in orange juice. These compounds may limit oxidative stress and inflammation, which are core mechanisms of the decline in muscle function and strength in old people as well as of frailty [Soysal et al, 2016; Soysal et al, 2017; Lauretani et al, 2008].* ´

Overall, it remains minor issues that should be addressed to improve this paper which is already of high quality.

Thank you very much for this kind comment and thorough review.

-----------------------------------------------------------

**Reviewer #3**: The authors present an investigation of associations between SSBs, ASBs and fruit juices, and frailty in the NHS. This is a well written, well argued, coherent manuscript which generates useful results with potential value to public health policy makers. I recommend publication of his paper with only a couple of very minor suggestions for improvement below.

Page 6, para 2: the FFQ is 'reasonably valid' - this is vague; what does reasonably valid mean?

Thank you. We have now removed these words and rephrased the sentence.

Page 8: ´*Previous research showed that, compared with multiple dietary records, 24-hour dietary recalls, and biomarkers of diet, the FFQ provides sufficient information to detect important associations with disease [23, 24] ´*.

Page 9, line 1: the number of participants included in the study belongs in the results, not the methods.

We have now indicated the number of participants in the Results section.

Page 17: ´*During 22 years of follow-up, we identified a total of 11,559 incident frailty cases among the 71,935 women of this study (Table 2)*. ´

-----------------------------------------------------------

**Reviewer #4:** This is a well-conducted study on the association between sweetened beverages and risk of frailty among older women using the Nurses´ Health Study (NHS) cohort. However, there are a few issues needing attention.

1) Competing risk. All the relative risks (RR) in table 2 were approximated by hazard ratios from the Cox proportional hazards models. However, as the outcome is frailty rather than all cause mortality, there is an issue of competing risk (from death). During the 22 years follow-up, potentially many participants (n=?) died. Therefore competing risk analyses need to be performed to derive the true HR/RR.

During follow-up, 3462 women died and were censored at their death time. We have used cause-specific hazard Cox model analysis to estimate the association between sweetened beverages and frailty. This is a type of competing risk analysis [Wang et al, 2016]. We have now better clarified this in the Statistical analysis section.

Page 11: ´*We used cause-specific proportional hazards models to calculate relative risks (RR) [Wang et al, 2016] ´*.

2) Many baseline variables were adjusted in table 2 as shown in the Multivariable model a and b. However, models were not adjusted for baseline co-morbidities which is not adequate. Although authors argued that the frailty outcome consists of some of the conditions, the baseline co-morbidities are different and need to be adjusted. In sensitivity analyses (supplementary table 5) the authors made some effort to address the issue but it is a bit patchy not systematic. It would be good to have a multivariable model c to additionally adjust these co-morbidities at baseline.

We had initially not included diseases into the model because the number of diseases is a component of the frailty outcome. Therefore, we adjusted for medication which is an indicator of the degree of multimorbidity and severity. However, as suggested by the reviewer, we have now added a model c that additionally includes heart disease, diabetes mellitus and cancer at baseline. Results have not materially changed after adjustment for baseline morbidity. Please, see the new tables.

3) Not clear whether SSBs, ASBs, orange juice and etc are mutually exclusive in the questionnaires. What if a person takes two more different types of drinks during the follow-ups? How did the authors adjust this potential overlap in the analyses?

The beverages included in the SSBs, ASBs and fruit juice categories were not mutually exclusive in the questionnaire. Participants were asked how often they consumed every specific beverage (i.e. caffeinated coals, non-caffeinated colas, apple juice, orange juice, etc.), and results were subsequently summed into beverage categories (SSBs, ASBs and fruit juices). The food frequency questionnaire was repeated in each cycle and the cumulative intake before frailty incidence was used to have a more precise estimate of the true habitual intake. We have now changed the description of the beverage categories to make this clearer.

Page 7-8: ‘*The consumptions of the following beverages were summed as SSBs: caffeinated and non-caffeinated colas [e.g., Coke, Pepsi, and other colas with sugar], other carbonated beverages with sugar [e.g., 7-Up], and noncarbonated sweetened beverages [e.g., Hawaiian Punch, lemonade, and other noncarbonated fruit drinks]. In addition, ASBs consisted of caffeinated, caffeine-free, and noncarbonated low-calorie or diet beverages. Fruit juices included orange juice, apple juice or cider, grapefruit juice, prune juice, and non-specified fruit juices*’.

In addition, to account for the simultaneous consumption of different types of beverages, we adjusted all models for the consumption of the types of beverages different to the exposure of interest. This provides the estimates for the association of one specific beverage with frailty, independently of the intake of other beverages. This has now been described clearer.

Page 12: ´*All models were mutually adjusted for the other types of beverages to obtain estimates for a beverage independent of the other beverages consumed*.’

4) Table 1, in the Dietary intake section, most variables appeared to be skewed with non-normal distributions therefore should be summarized as median and IQR rather than mean and SD.

Thank you. The dietary intakes for the categories of sweetened beverage intake in Table 1 are now presented as median and IQR.

-----------------------------------------------------------
